# Supplementary material for: Shade-induced nuclear localization of PIF7 is regulated by phosphorylation and 14-3-3 proteins in Arabidopsis
Source: eLife. 2018 Jun 21;7:e31636. doi: 10.7554/eLife.31636 (PMC6037483; doi:10.7554/eLife.31636)
Supplement: Figure 1—source data 1. [file elife-31636-fig1-data1.docx]

**Figure 1-source data 1.** Source files for the ratios of the nuclear and cytoplasmic signal intensities in figure 1b.

| Replicate | WL | | | | | | | | | | | |
| --- | --- | --- | --- | --- | --- | --- | --- | --- | --- | --- | --- | --- |
|  | 6# | | | | 14# | | | | GFP | | | |
|  | Total | Nucl | Cyto | Ratio | Total | Nucl | Cyto | Ratio | Total | Nucl | Cyto | Ratio |
| 1 | 111.29 | 34.54 | 76.75 | 0.45 | 78.53 | 35.62 | 42.91 | 0.83 | 50.91 | 26.20 | 24.71 | 1.06 |
| 2 | 89.68 | 36.61 | 53.07 | 0.69 | 110.74 | 52.76 | 57.98 | 0.91 | 107.81 | 75.82 | 31.99 | 2.37 |
| 3 | 97.20 | 41.66 | 55.54 | 0.75 | 96.66 | 47.09 | 49.57 | 0.95 | 91.54 | 64.05 | 27.49 | 2.33 |
| 4 | 117.72 | 39.76 | 77.96 | 0.51 | 38.10 | 16.33 | 21.77 | 0.75 | 70.92 | 38.39 | 32.53 | 1.18 |
| 5 | 107.46 | 42.33 | 65.13 | 0.65 | 76.38 | 33.47 | 42.91 | 0.78 | 102.83 | 63.28 | 39.55 | 1.60 |
| 6 | 76.65 | 6.33 | 70.32 | 0.09 | 86.34 | 40.90 | 45.44 | 0.90 | 118.75 | 82.10 | 36.65 | 2.24 |
| 7 | 82.46 | 31.56 | 50.90 | 0.62 | 137.73 | 68.17 | 69.56 | 0.98 | 54.43 | 28.51 | 25.92 | 1.10 |
| 8 | 83.72 | 25.17 | 58.55 | 0.43 | 101.47 | 48.62 | 52.85 | 0.92 | 78.73 | 45.08 | 33.65 | 1.34 |
| 9 | 94.13 | 35.30 | 58.83 | 0.60 | 71.10 | 30.00 | 41.10 | 0.73 | 48.91 | 22.33 | 26.58 | 0.84 |
| 10 | 89.51 | 32.86 | 56.65 | 0.58 | 66.54 | 27.40 | 39.14 | 0.70 | 96.34 | 63.46 | 32.88 | 1.93 |
| Average | | | | 0.54 | Average | | | 0.85 | Average | | | 1.60 |
| SD | | | | 0.19 | SD | | | 0.10 | SD | | | 0.58 |

| Replicate | 5 min SH | | | | | | | | | | | |
| --- | --- | --- | --- | --- | --- | --- | --- | --- | --- | --- | --- | --- |
|  | 6# | | | | 14# | | | | GFP | | | |
|  | Total | Nucl | Cyto | Ratio | Total | Nucl | Cyto | Ratio | Total | Nucl | Cyto | Ratio |
| 1 | 63.73 | 47.80 | 15.93 | 3.00 | 92.88 | 73.77 | 19.11 | 3.86 | 91.25 | 58.66 | 32.59 | 1.80 |
| 2 | 77.17 | 57.28 | 19.89 | 2.88 | 152.32 | 123.08 | 29.24 | 4.21 | 60.34 | 42.38 | 17.96 | 2.36 |
| 3 | 59.70 | 46.55 | 13.15 | 3.54 | 100.89 | 79.78 | 21.11 | 3.78 | 95.83 | 61.85 | 33.98 | 1.82 |
| 4 | 73.34 | 56.08 | 17.26 | 3.25 | 102.68 | 85.13 | 17.55 | 4.85 | 72.65 | 32.51 | 40.14 | 0.81 |
| 5 | 99.17 | 78.64 | 20.53 | 3.83 | 73.73 | 57.98 | 15.75 | 3.68 | 100.06 | 63.14 | 36.92 | 1.71 |
| 6 | 130.02 | 106.76 | 23.26 | 4.59 | 110.79 | 89.15 | 21.64 | 4.12 | 80.26 | 48.66 | 31.60 | 1.54 |
| 7 | 58.74 | 44.09 | 14.65 | 3.01 | 101.2 | 80.03 | 21.17 | 3.78 | 78.49 | 57.22 | 21.27 | 2.69 |
| 8 | 57.14 | 44.30 | 12.84 | 3.45 | 81.26 | 63.00 | 18.26 | 3.45 | 123.73 | 85.54 | 38.19 | 2.24 |
| 9 | 81.80 | 66.13 | 15.67 | 4.22 | 110.44 | 88.35 | 22.09 | 4.00 | 90.05 | 53.74 | 36.31 | 1.48 |
| 10 | 130.23 | 102.16 | 28.07 | 3.64 | 70.99 | 54.86 | 16.13 | 3.40 | 57.22 | 29.17 | 28.05 | 1.04 |
| Average | | | | 3.54 | Average | | | 3.91 | Average | | | 1.75 |
| SD | | | | 0.55 | SD | | | 0.42 | SD | | | 0.58 |

| Replicate | 15 min SH | | | | | | | | | | | |
| --- | --- | --- | --- | --- | --- | --- | --- | --- | --- | --- | --- | --- |
|  | 6# | | | | 14# | | | | GFP | | | |
|  | Total | Nucl | Cyto | Ratio | Total | Nucl | Cyto | Ratio | Total | Nucl | Cyto | Ratio |
| 1 | 131.86 | 119.35 | 12.51 | 9.54 | 47.64 | 35.14 | 12.50 | 2.81 | 72.52 | 54.61 | 17.91 | 3.05 |
| 2 | 172.34 | 155.51 | 16.83 | 9.24 | 66.89 | 54.86 | 12.03 | 4.56 | 83.06 | 43.51 | 39.55 | 1.10 |
| 3 | 121.80 | 105.94 | 15.86 | 6.68 | 115.31 | 104.33 | 10.98 | 9.50 | 81.15 | 48.56 | 32.59 | 1.49 |
| 4 | 88.66 | 79.75 | 8.91 | 8.95 | 75.86 | 67.22 | 8.64 | 7.78 | 83.05 | 42.93 | 40.12 | 1.07 |
| 5 | 119.96 | 106.14 | 13.82 | 7.68 | 121.20 | 103.24 | 17.96 | 5.75 | 61.07 | 36.93 | 24.14 | 1.53 |
| 6 | 81.43 | 73.42 | 8.01 | 9.17 | 44.28 | 34.03 | 10.25 | 3.32 | 59.94 | 38.61 | 21.33 | 1.81 |
| 7 | 77.81 | 68.00 | 9.81 | 6.93 | 123.37 | 104.65 | 18.72 | 5.59 | 68.17 | 42.25 | 25.92 | 1.63 |
| 8 | 132.63 | 117.10 | 15.53 | 7.54 | 72.98 | 64.10 | 8.88 | 7.22 | 126.44 | 89.79 | 36.65 | 2.45 |
| 9 | 138.24 | 126.71 | 11.53 | 10.99 | 119.42 | 104.79 | 14.63 | 7.16 | 94.42 | 56.95 | 37.47 | 1.52 |
| 10 | 65.69 | 58.31 | 7.38 | 7.90 | 116.56 | 103.49 | 13.07 | 7.92 | 71.34 | 39.49 | 31.85 | 1.24 |
| Average | | | | 8.46 | Average | | | 6.16 | Average | | | 1.69 |
| SD | | | | 1.34 | SD | | | 2.14 | SD | | | 0.62 |

| Replicate | 25 min SH | | | | | | | | | | | |
| --- | --- | --- | --- | --- | --- | --- | --- | --- | --- | --- | --- | --- |
|  | 6# | | | | 14# | | | | GFP | | | |
|  | Total | Nucl | Cyto | Ratio | Total | Nucl | Cyto | Ratio | Total | Nucl | Cyto | Ratio |
| 1 | 164.70 | 154.40 | 10.30 | 14.99 | 100.64 | 93.66 | 6.98 | 13.42 | 57.40 | 21.30 | 36.10 | 0.59 |
| 2 | 30.45 | 27.96 | 2.49 | 11.23 | 90.77 | 86.12 | 4.65 | 18.52 | 64.10 | 36.47 | 27.63 | 1.32 |
| 3 | 26.80 | 23.05 | 3.75 | 6.15 | 95.80 | 90.37 | 5.43 | 16.64 | 77.77 | 47.03 | 30.74 | 1.53 |
| 4 | 106.36 | 91.10 | 15.26 | 5.97 | 73.63 | 68.09 | 5.54 | 12.29 | 77.83 | 46.32 | 31.51 | 1.47 |
| 5 | 205.91 | 193.40 | 12.51 | 15.46 | 81.19 | 76.13 | 5.06 | 15.04 | 83.51 | 50.37 | 33.14 | 1.52 |
| 6 | 104.49 | 96.94 | 7.55 | 12.84 | 105.52 | 99.19 | 6.33 | 15.67 | 103.06 | 65.45 | 37.61 | 1.74 |
| 7 | 95.47 | 87.95 | 7.52 | 11.70 | 104.55 | 98.78 | 5.77 | 17.12 | 107.97 | 67.07 | 40.90 | 1.64 |
| 8 | 106.45 | 98.49 | 7.96 | 12.37 | 78.70 | 73.35 | 5.35 | 13.71 | 58.21 | 32.68 | 25.53 | 1.28 |
| 9 | 103.09 | 89.89 | 13.20 | 6.81 | 92.41 | 87.05 | 5.36 | 16.24 | 69.02 | 35.02 | 34.00 | 1.03 |
| 10 | 47.15 | 44.09 | 3.06 | 14.41 | 139.41 | 130.74 | 8.67 | 15.08 | 73.19 | 41.09 | 32.10 | 1.28 |
| Average | | | | 11.19 | Average | | | 15.37 | Average | | | 1.34 |
| SD | | | | 3.64 | SD | | | 1.88 | SD | | | 0.33 |

| Replicate | 45 min SH | | | | | | | | | | | |
| --- | --- | --- | --- | --- | --- | --- | --- | --- | --- | --- | --- | --- |
|  | 6# | | | | 14# | | | | GFP | | | |
|  | Total | Nucl | Cyto | Ratio | Total | Nucl | Cyto | Ratio | Total | Nucl | Cyto | Ratio |
| 1 | 85.44 | 78.32 | 7.12 | 11.00 | 89.39 | 85.65 | 3.74 | 22.90 | 59.45 | 32.67 | 26.78 | 1.22 |
| 2 | 76.82 | 70.00 | 6.82 | 10.27 | 91.67 | 86.07 | 5.60 | 15.37 | 147.37 | 113.65 | 33.72 | 3.37 |
| 3 | 106.4 | 99.95 | 6.45 | 15.50 | 136.37 | 128.02 | 8.35 | 15.33 | 88.01 | 59.34 | 28.67 | 2.07 |
| 4 | 139.51 | 131.27 | 8.24 | 15.93 | 92.33 | 83.57 | 8.76 | 9.54 | 92.20 | 56.60 | 35.60 | 1.59 |
| 5 | 81.27 | 76.93 | 4.34 | 17.73 | 82.88 | 78.75 | 4.13 | 19.07 | 65.71 | 35.43 | 30.28 | 1.17 |
| 6 | 135.45 | 127.44 | 8.01 | 15.91 | 122.45 | 116.63 | 5.82 | 20.04 | 118.50 | 89.17 | 29.33 | 3.04 |
| 7 | 113.3 | 106.85 | 6.45 | 16.57 | 161.66 | 154.02 | 7.64 | 20.16 | 50.49 | 31.29 | 19.20 | 1.63 |
| 8 | 90.01 | 82.31 | 7.70 | 10.69 | 110.17 | 105.06 | 5.11 | 20.56 | 74.28 | 45.49 | 28.79 | 1.58 |
| 9 | 146.42 | 138.24 | 8.18 | 16.90 | 73.88 | 69.68 | 4.20 | 16.59 | 77.89 | 56.01 | 21.88 | 2.56 |
| 10 | 65.85 | 62.05 | 3.80 | 16.33 | 126.94 | 120.13 | 6.81 | 17.64 | 77.06 | 52.28 | 24.78 | 2.11 |
| Average | | | | 14.68 | Average | | | 17.72 | Average | | | 2.03 |
| SD | | | | 2.85 | SD | | | 3.76 | SD | | | 0.75 |
