# Supplementary material for: Shade-induced nuclear localization of PIF7 is regulated by phosphorylation and 14-3-3 proteins in Arabidopsis
Source: eLife. 2018 Jun 21;7:e31636. doi: 10.7554/eLife.31636 (PMC6037483; doi:10.7554/eLife.31636)
Supplement: Figure 5—source data 1. [file elife-31636-fig5-data1.docx]

**Figure 5-source data 1.** Source files for the ratios of the nuclear and cytoplasmic signal intensities in and figure 5b and figure 5e.

| Replicate | WL | | | | | | | |
| --- | --- | --- | --- | --- | --- | --- | --- | --- |
|  | R18 | | | | R18(Lys) | | | |
|  | Total | Nucl | Cyto | Ratio | Total | Nucl | Cyto | Ratio |
| 1 | 121.07 | 56.67 | 64.40 | 0.88 | 54.59 | 24.76 | 29.83 | 0.83 |
| 2 | 65.79 | 20.42 | 45.37 | 0.45 | 87.34 | 38.82 | 48.52 | 0.80 |
| 3 | 116.48 | 51.77 | 64.71 | 0.80 | 60.28 | 23.07 | 37.21 | 0.62 |
| 4 | 45.29 | 13.17 | 32.12 | 0.41 | 94.29 | 37.83 | 56.46 | 0.67 |
| 5 | 88.26 | 34.44 | 53.82 | 0.64 | 74.87 | 34.40 | 40.47 | 0.85 |
| 6 | 73.02 | 24.66 | 48.36 | 0.51 | 80.30 | 35.69 | 44.61 | 0.80 |
| 7 | 63.77 | 31.07 | 32.70 | 0.95 | 60.13 | 24.55 | 35.58 | 0.69 |
| 8 | 116.66 | 43.75 | 72.91 | 0.60 | 93.32 | 34.63 | 58.69 | 0.59 |
| 9 | 84.50 | 30.68 | 53.82 | 0.57 | 83.54 | 41.35 | 42.19 | 0.98 |
| 10 | 107.04 | 45.17 | 61.87 | 0.73 | 102.72 | 46.28 | 56.44 | 0.82 |
| Average | 0.65 | | | | 0.77 | | | |
| SD | 0.18 | | | | 0.12 | | | |

| Replicate | 2 min SH | | | | | | | |
| --- | --- | --- | --- | --- | --- | --- | --- | --- |
|  | R18 | | | | R18(Lys) | | | |
|  | Total | Nucl | Cyto | Ratio | Total | Nucl | Cyto | Ratio |
| 1 | 42.48 | 33.81 | 8.67 | 3.90 | 104.22 | 82.86 | 21.36 | 3.88 |
| 2 | 63.62 | 51.17 | 12.45 | 4.11 | 66.69 | 51.43 | 15.26 | 3.37 |
| 3 | 158.58 | 127.24 | 31.34 | 4.06 | 183.30 | 144.63 | 38.67 | 3.74 |
| 4 | 69.11 | 54.56 | 14.55 | 3.75 | 94.40 | 74.23 | 20.17 | 3.68 |
| 5 | 57.73 | 46.16 | 11.57 | 3.99 | 59.56 | 44.78 | 14.78 | 3.03 |
| 6 | 78.77 | 64.29 | 14.48 | 4.44 | 72.97 | 59.63 | 13.34 | 4.47 |
| 7 | 35.37 | 28.45 | 6.92 | 4.11 | 119.88 | 98.24 | 21.64 | 4.54 |
| 8 | 70.21 | 56.39 | 13.82 | 4.08 | 63.57 | 51.71 | 11.86 | 4.36 |
| 9 | 60.95 | 49.21 | 11.74 | 4.19 | 76.83 | 58.80 | 18.04 | 3.26 |
| 10 | 69.58 | 57.11 | 12.47 | 4.58 | 80.56 | 64.35 | 16.21 | 3.97 |
| Average | 4.12 | | | | 3.83 | | | |
| SD | 0.24 | | | | 0.52 | | | |

| Replicate | 10 min SH | | | | | | | |
| --- | --- | --- | --- | --- | --- | --- | --- | --- |
|  | R18 | | | | R18(Lys) | | | |
|  | Total | Nucl | Cyto | Ratio | Total | Nucl | Cyto | Ratio |
| 1 | 89.42 | 79.78 | 9.64 | 8.26 | 60.32 | 52.07 | 8.25 | 6.31 |
| 2 | 143.94 | 128.38 | 15.56 | 8.25 | 104.54 | 92.51 | 12.03 | 7.69 |
| 3 | 77.17 | 66.05 | 11.12 | 5.94 | 135.21 | 117.25 | 17.96 | 6.53 |
| 4 | 111.41 | 97.09 | 14.32 | 6.78 | 51.05 | 43.91 | 7.14 | 6.15 |
| 5 | 76.80 | 68.65 | 8.15 | 8.42 | 107.10 | 94.26 | 12.84 | 7.34 |
| 6 | 83.54 | 74.70 | 8.84 | 8.45 | 80.62 | 68.82 | 11.80 | 5.83 |
| 7 | 90.91 | 78.92 | 11.99 | 6.58 | 76.72 | 67.61 | 9.11 | 7.42 |
| 8 | 66.47 | 58.28 | 8.19 | 7.12 | 68.92 | 58.48 | 10.44 | 5.60 |
| 9 | 168.88 | 152.84 | 16.04 | 9.53 | 65.29 | 52.83 | 12.46 | 4.24 |
| 10 | 105.29 | 92.42 | 12.87 | 7.18 | 77.29 | 68.89 | 8.40 | 8.20 |
| Average | 7.65 | | | | 6.53 | | | |
| SD | 1.10 | | | | 1.17 | | | |

| Replicate | WL | | | | | | | |
| --- | --- | --- | --- | --- | --- | --- | --- | --- |
|  | 14-3-3 double | | | | Col-0 | | | |
|  | Total | Nucl | Cyto | Ratio | Total | Nucl | Cyto | Ratio |
| 1 | 105.40 | 45.17 | 60.23 | 0.75 | 67.37 | 26.04 | 41.33 | 0.63 |
| 2 | 83.71 | 35.04 | 48.67 | 0.72 | 90.04 | 37.69 | 52.35 | 0.72 |
| 3 | 111.12 | 37.04 | 74.08 | 0.50 | 114.79 | 51.37 | 63.42 | 0.81 |
| 4 | 43.15 | 16.18 | 26.97 | 0.60 | 96.84 | 38.15 | 58.69 | 0.65 |
| 5 | 60.00 | 20.53 | 39.47 | 0.52 | 80.60 | 33.19 | 47.41 | 0.70 |
| 6 | 50.69 | 20.52 | 30.17 | 0.68 | 111.05 | 38.47 | 72.58 | 0.53 |
| 7 | 21.28 | 7.46 | 13.82 | 0.54 | 116.32 | 44.07 | 72.25 | 0.61 |
| 8 | 101.99 | 36.19 | 65.80 | 0.55 | 88.17 | 34.41 | 53.76 | 0.64 |
| 9 | 50.70 | 13.96 | 36.74 | 0.38 | 92.15 | 44.90 | 47.26 | 0.95 |
| 10 | 82.81 | 41.61 | 41.20 | 1.01 | 60.35 | 23.10 | 37.25 | 0.62 |
| Average | 0.63 | | | | 0.69 | | | |
| SD | 0.18 | | | | 0.12 | | | |

| Replicate | 2 min SH | | | | | | | |
| --- | --- | --- | --- | --- | --- | --- | --- | --- |
|  | 14-3-3 double | | | | Col-0 | | | |
|  | Total | Nucl | Cyto | Ratio | Total | Nucl | Cyto | Ratio |
| 1 | 171.18 | 143.12 | 28.06 | 5.10 | 87.57 | 66.21 | 21.36 | 3.10 |
| 2 | 74.23 | 62.54 | 11.69 | 5.35 | 133.06 | 104.69 | 28.37 | 3.69 |
| 3 | 76.29 | 63.23 | 13.06 | 4.84 | 100.55 | 81.79 | 18.76 | 4.36 |
| 4 | 91.97 | 77.60 | 14.37 | 5.40 | 81.90 | 66.15 | 15.75 | 4.20 |
| 5 | 81.15 | 68.14 | 13.01 | 5.24 | 89.47 | 69.85 | 19.62 | 3.56 |
| 6 | 75.53 | 63.46 | 12.07 | 5.26 | 50.41 | 37.84 | 12.57 | 3.01 |
| 7 | 88.31 | 74.49 | 13.82 | 5.39 | 71.15 | 56.92 | 14.23 | 4.00 |
| 8 | 82.36 | 68.26 | 14.10 | 4.84 | 67.52 | 52.81 | 14.71 | 3.59 |
| 9 | 103.73 | 87.82 | 15.91 | 5.52 | 61.57 | 47.98 | 13.59 | 3.53 |
| 10 | 149.67 | 124.89 | 24.78 | 5.04 | 45.33 | 37.21 | 8.12 | 4.58 |
| Average | 5.20 | | | | 3.76 | | | |
| SD | 0.24 | | | | 0.52 | | | |

| Replicate | 10 min SH | | | | | | | |
| --- | --- | --- | --- | --- | --- | --- | --- | --- |
|  | 14-3-3 double | | | | Col-0 | | | |
|  | Total | Nucl | Cyto | Ratio | Total | Nucl | Cyto | Ratio |
| 1 | 72.94 | 65.28 | 7.66 | 8.52 | 86.51 | 76.69 | 9.82 | 7.81 |
| 2 | 62.23 | 55.52 | 6.71 | 8.27 | 75.31 | 63.28 | 12.03 | 5.26 |
| 3 | 85.80 | 77.20 | 8.60 | 8.98 | 94.80 | 81.41 | 13.39 | 6.08 |
| 4 | 44.97 | 39.68 | 5.29 | 7.50 | 53.06 | 47.32 | 5.74 | 8.25 |
| 5 | 94.48 | 86.29 | 8.19 | 10.54 | 119.84 | 105.19 | 14.65 | 7.18 |
| 6 | 45.78 | 41.40 | 4.38 | 9.45 | 57.23 | 48.96 | 8.27 | 5.92 |
| 7 | 31.04 | 27.02 | 4.02 | 6.72 | 90.12 | 79.58 | 10.54 | 7.55 |
| 8 | 58.50 | 52.29 | 6.21 | 8.42 | 109.89 | 91.93 | 17.96 | 5.12 |
| 9 | 76.81 | 68.49 | 8.32 | 8.23 | 124.39 | 110.81 | 13.58 | 8.16 |
| 10 | 79.34 | 71.96 | 7.38 | 9.75 | 70.40 | 61.73 | 8.67 | 7.12 |
| Average | 8.64 | | | | 6.85 | | | |
| SD | 1.10 | | | | 1.17 | | | |
