# Supplementary material for: Shade-induced nuclear localization of PIF7 is regulated by phosphorylation and 14-3-3 proteins in Arabidopsis
Source: eLife. 2018 Jun 21;7:e31636. doi: 10.7554/eLife.31636 (PMC6037483; doi:10.7554/eLife.31636)
Supplement: Figure 5—source data 2. [file elife-31636-fig5-data2.docx]

**Figure 5-source data 2.** Source files for the ratios of phosphorylated PIF7(PIF7[p]) relative to total PIF7 proteins in figure supplement 1.

| Replicate | Treatment | Time point | PIF7[p] | Total PIF7 | Ratio | Normalized |
| --- | --- | --- | --- | --- | --- | --- |
| 1 | R18 | 0 min SH | 219163 | 375906 | 0.58 | 1.00 |
|  |  | 2 min SH | 124560 | 350795 | 0.36 | 0.62 |
|  |  | 5 min SH | 78179 | 333671 | 0.23 | 0.40 |
|  | R18(Lys) | 0 min SH | 154196 | 314459 | 0.49 | 1.00 |
|  |  | 2 min SH | 144047 | 351335 | 0.41 | 0.84 |
|  |  | 5 min SH | 147973 | 469039 | 0.32 | 0.65 |
| 2 | R18 | 0 min SH | 133836 | 257376 | 0.52 | 1.00 |
|  |  | 2 min SH | 100240 | 323355 | 0.31 | 0.60 |
|  |  | 5 min SH | 83037 | 307544 | 0.27 | 0.52 |
|  | R18(Lys) | 0 min SH | 146439 | 287740 | 0.51 | 1.00 |
|  |  | 2 min SH | 172919 | 394501 | 0.44 | 0.86 |
|  |  | 5 min SH | 96926 | 334132 | 0.29 | 0.57 |
| 3 | R18 | 0 min SH | 132075 | 281501 | 0.47 | 1.00 |
|  |  | 2 min SH | 73651 | 212025 | 0.35 | 0.74 |
|  |  | 5 min SH | 59473 | 291651 | 0.20 | 0.43 |
|  | R18(Lys) | 0 min SH | 200889 | 398718 | 0.50 | 1.00 |
|  |  | 2 min SH | 158095 | 335054 | 0.47 | 0.94 |
|  |  | 5 min SH | 91433 | 241729 | 0.38 | 0.76 |

| Replicate | Background | Time point | PIF7[p] | Total PIF7 | Ratio | Normalized |
| --- | --- | --- | --- | --- | --- | --- |
| 1 | 14-3-3 double | 0 min SH | 137550 | 360624 | 0.38 | 1.00 |
|  |  | 2 min SH | 47927 | 247301 | 0.19 | 0.51 |
|  |  | 5 min SH | 55208 | 427307 | 0.13 | 0.34 |
|  | Col-0 | 0 min SH | 95432 | 265562 | 0.36 | 1.00 |
|  |  | 2 min SH | 117066 | 382568 | 0.31 | 0.85 |
|  |  | 5 min SH | 110952 | 447465 | 0.25 | 0.69 |
| 2 | 14-3-3 double | 0 min SH | 184073 | 503700 | 0.37 | 1.00 |
|  |  | 2 min SH | 73896 | 332865 | 0.22 | 0.61 |
|  |  | 5 min SH | 58808 | 349830 | 0.17 | 0.46 |
|  | Col-0 | 0 min SH | 169088 | 435688 | 0.39 | 1.00 |
|  |  | 2 min SH | 191673 | 522840 | 0.37 | 0.94 |
|  |  | 5 min SH | 118274 | 522874 | 0.23 | 0.58 |
| 3 | 14-3-3 double | 0 min SH | 131552 | 314748 | 0.42 | 1.00 |
|  |  | 2 min SH | 114731 | 440595 | 0.26 | 0.62 |
|  |  | 5 min SH | 48139 | 272897 | 0.18 | 0.42 |
|  | Col-0 | 0 min SH | 134610 | 275528 | 0.49 | 1.00 |
|  |  | 2 min SH | 121654 | 306510 | 0.40 | 0.81 |
|  |  | 5 min SH | 135255 | 368041 | 0.37 | 0.75 |
