# Supplementary material for: Shade-induced nuclear localization of PIF7 is regulated by phosphorylation and 14-3-3 proteins in Arabidopsis
Source: eLife. 2018 Jun 21;7:e31636. doi: 10.7554/eLife.31636 (PMC6037483; doi:10.7554/eLife.31636)
Supplement: Supplementary file 1. [file elife-31636-supp1.docx]

Supplementary file 1. Primers used in this study.

| 14-3-3 λ-F | ATCTTCCAGAGATGGATCCATGGCGGCGACATTAG |
| --- | --- |
| 14-3-3 λ-R | GCCGTTCGACGATGTCGACTCAGGCCTCGTCCATC |
| 14-3-3 κ-F | ATCTTCCAGAGATGGATCCATGGCGACGACCTTAAGCAGAGATC |
| 14-3-3 κ-R | GCCGTTCGACGATGTCGACTCAGGCCTCATCCATCTGCTCCTGC |
| PIF7-F | GGATCCATGTCGAATTATGGAGTTAAAGAG |
| PIF7-R | GAGCTCATCTCTTTTCTCATGATTCGAAGA |
| qRT-14-3-3 λ-F | CGAGTGAGTCTAAGGTCT |
| qRT-14-3-3 λ-R | GTATCTTCAGCAGCAGTT |
| qRT-YUCCA8-F | TGAAACAAAACAACCCACGA |
| qRT-YUCCA8-R | TTGATTCGCTTTGGGTCTTC |
| qRT-IAA19-F | GACGTCGTCGGGTAGTAATAGTGA |
| qRT-IAA19-R | GATGAAACGACGCCGCTTT |
| SALK_071097-LP | AAGCGTTTCATCAGTCTCGAG |
| SALK_071097-RP | AGGCTTGGTTTGGCTCTTAAC |
| CS482153-LP | GATCAGATGCGAGTCAAGGAG |
| CS482153-RP | CGATTAAGCTTTGCATTCGAG |
| SALK _148929C-LP | TCAGAGTGTGTTAATCACGCG |
| SALK_148929C-RP | ATACAAGATGGGGCCAATAGG |
| SALK _075219C-LP | TAAGGCTTGAGCAGAGTCGAG |
| SALK_075219C-RP | TTCTTTCTTTTTCGCTGCTTG |
| TSK108-PIF7-F | CCGGAATTCTTCGAATTATGGAGTTAAAGAG |
| TSK108-PIF7-R | GGCGGATCCCTAATCTCTTTTCTCATGAT |
| PIF7(S139A-S141A)-F | CAGAGACTATTTCCGCGCTGGAGCGGAAACTCAAGATAC |
| PIF7(S139A-S141A)-R | gtatcttgagtttccgCtccagCgcggaaatagtctctg |
| PIF7(S139D-S141D)-F | CAGAGACTATTTCCGCGACGGAGACGAAACTCAAGATAC |
| PIF7(S139D-S141D)-R | GTATCTTGAGTTTCGTCTCCGTCGCGGAAATAGTCTCTG |
| PIF7(S78A-S80A)-F | tgagagcaaggatggaGcttgtGcaagaaaacgcggtta |
| PIF7(S78A-S80A)-R | taaccgcgttttcttgCacaagCtccatccttgctctca |
| PIF7(S78D-S80D)-F | TGAGAGCAAGGATGGAGACTGTGACAGAAAACGCGGTTA |
| PIF7(S78D-S80D)-R | TAACCGCGTTTTCTGTCACAGTCTCCATCCTTGCTCTCA |
| PIF7(S125D)-F | CTTTTGAATCCGGTCGGGACTTGAAGACAGCTAGA |
| PIF7(S125D)-R | TCTAGCTGTCTTCAAGTCCCGACCGGATTCAAAAG |
| PIF7(S125A)-F | cttttgaatccggtcggGCcttgaagacagctaga |
| PIF7(S125A)-R | tctagctgtcttcaagGCccgaccggattcaaaag |
| PIF7△-R | GAAATAGTCTCTGTCTCCGGTTGAAACTCAAGATACT |
| PIF7△-F | GACAGAGACTATTTCGAAACTCAAGATACTGAAGGAGAT |
| 14-3-3 χ-F | CGCGGATCCATGGCGACACCAGGAGCTTC |
| 14-3-3 χ-R | ACGCGTCGACGGATTGTTGCTCGTCAGCG |
| 14-3-3 γ-F | CGCGGATCCATGTCTTCTGATTCGTCCCGG |
| 14-3-3 γ-R | ACGCGTCGACCTGCGAAGGTGGTGGTTGG |
| 14-3-3 μ-F | CGCGGATCCATGGGTTCTGGAAAAGAGCGT |
| 14-3-3 μ-R | ACGCGTCGACCTCTGCATCGTCTCCACCAG |
| 14-3-3 ε-F | CGCGGATCCATGGAGAATGAGAGGGAAAAGCA |
| 14-3-3 ε-R | ACGCGTCGACGTTCTCATCTTGAGGCTCATCAGC |
